# HIV proviral transcription and infectivity are enhanced by neddylation

**DOI:** 10.1128/jvi.00968-25

**Published:** 2025-09-30

**Authors:** Cristina C. Vaca, Hannah Hudson, Isabelle Clerc, Chisu Song, Richard T. D'Aquila

**Affiliations:** 1 Department of Medicine, Division of Infectious Diseases, Feinberg School of Medicine, Northwestern University 273158 , Chicago, Illinois, USA; Icahn School of Medicine at Mount Sinai, New York, New York, USA

**Keywords:** HIV, HIV latency, HIV reactivation, neddylation, Cullin-RING ligases (CRLs), HIV latency-reversing agents (LRAs), TNFα, PMA and ionomycin, JQ1, NF-kB, inhibitor of kB alpha (IkBα), latency-reversing agents, strategies for ART-free remission of HIV infection, HIV cure research, shock and kill, APOBEC3G

## Abstract

A spreading infection can quickly restart from the persistent reservoir of cells harboring HIV proviruses if antiretroviral therapy (ART) is stopped. HIV transcription can also be increased by latency-reversing agents (LRAs) during ART. Neddylation is a post-translational modification that activates Cullin-RING ligases (CRLs), which ubiquitinate several proteins that regulate HIV transcription and infectivity, marking these regulators for proteasomal degradation. We studied how inhibiting neddylation affects HIV gene expression *ex vivo* after three LRAs: TNFα, PMA and ionomycin, and JQ1. In provirus-containing T cells, broad inhibition of CRL-mediated ubiquitination using MLN4924 (MLN) with the above LRAs reduced HIV transcriptional initiation, decreased virus production, and diminished virion infectivity by increasing A3G incorporation. Decreased degradation of inhibitor of kB alpha (IkBα) was implicated in reducing LRA-stimulated HIV transcription. MLN also decreased HIV reactivation after PMA and ionomycin treatment of CD4+ T cells from ART-suppressed people living with HIV. Results indicate that neddylation can enhance HIV proviral transcription and reactivated virion infectivity.

**IMPORTANCE:**

Results indicate that neddylation contributes to reactivating HIV provirus transcription and antigen expression, as well as enhancing infectivity of resulting virions. This suggests hypotheses to test in the future that may inform a novel strategy for research to enable antiretroviral therapy (ART)-free remission of HIV infection, including whether inhibiting neddylation when ART stops reduces spontaneous provirus reactivation and increases virus A3G content to help control HIV rebound from latent reservoirs post-ART.

## INTRODUCTION

Current antiretroviral therapy (ART) durably suppresses HIV plasma viral load below the limit of detection in people living with HIV (PLWH). Nevertheless, integrated HIV proviruses persist in some long-lived infected cells, including memory CD4+ T cells, even after extended periods of ART treatment (1, 2). If ART is stopped, virions produced from some of these provirus-containing cells can quickly restart a spreading infection and lead to viremia rebound. Research on controlling or eradicating persistent proviruses to prevent viremia rebound after ART cessation has focused on enhancing provirus transcription during ART with HIV latency reversing agents (LRAs). Success has been limited. This encourages further characterization of provirus transcriptional control to better inform strategies to prevent recurrent viremia after stopping ART. Here, we study whether neddylation contributes to increased HIV provirus transcription and antigen expression following tumor necrosis factor alpha (TNFα), phorbol myristate acetate and ionomycin (PMAi), and BET-bromodomain inhibitor JQ1, which are each well documented to increase transcription and virus production from provirus-containing ACH2 and J-Lat cells (3).

Neddylation is a post-translational modification that attaches a ubiquitin-like protein, Nedd8, to lysine residues (4). This modification to any of the eight Cullins in the many Cullin-RING Ligases (CRLs) is necessary to activate the enzymatic function of virtually all CRLs. This enables a CRL to polyubiquitinate its substrate protein, directing it for proteasomal degradation (5). Several cellular proteins that are degraded after activation of CRL ubiquitination have been reported to affect HIV transcription, including inhibitors of NF-kB (6–10). Therefore, we studied the effects of broadly inhibiting CRL-mediated degradation on LRA-induced provirus transcription and HIV protein expression *ex vivo* in provirus-containing T cells.

In this work, we used a small molecule Nedd8-activating-enzyme inhibitor, MLN4924 (also known as pevonedistat, referred to here as MLN), to inhibit CRL activity. MLN was developed as an adjunctive therapy for certain cancers and was shown to be safe in multiple clinical trials (11–17). Among its broad effects, MLN has been shown to block degradation of several proteins critical for *de novo* HIV infections in T cells and monocyte-derived macrophages, including those co-opted by HIV accessory gene products Vif, Vpr, and Vpu (18–22). For example, previous work showed that MLN reduced HIV virion infectivity and spread through a culture after *de novo* infection by protecting the anti-viral host factor APOBEC3G (A3G) from Cullin-5 (CUL5)-mediated degradation by HIV Vif (18, 19). However, prior works were limited to *de novo* infections of HIV and did not study the effects of inhibiting neddylation on infectivity of viruses reactivated from cells persistently harboring proviruses, the effects on provirus transcription, nor the subsequent production of virus from provirus-containing cells.

Here, we investigated the effect of MLN on provirus-containing cells induced to express and release HIV following treatment with three LRAs: TNFα, PMAi, and JQ1. Given previous data from cancer studies that MLN can reduce NF-kB signaling (23–26), we studied whether inhibiting neddylation reduced HIV transcription in addition to evaluating its effects on A3G. We characterized how MLN affected HIV transcription following these LRAs in ACH2 and J-Lat cells. Effects on HIV transcription in CD4+ T cells from ART-suppressed PLWH were also studied after treatment with PMAi *ex vivo*. The amount and infectivity of viruses produced from provirus-containing T-cell lines after each of the three LRAs was also assessed. Results show that inhibiting neddylation limited increases in provirus transcriptional initiation caused by each of these three different LRAs and suggested that MLN decreased the degradation of an inhibitor of NF-kB. MLN also decreased the production and infectivity of Vif+ viruses reactivated from ACH2 cells. These results indicate that neddylation enhances provirus transcription and virus spread caused by these LRAs.

## RESULTS

### Inhibiting neddylation reduced HIV expression in provirus-containing cells after LRAs

**Fig 1:**
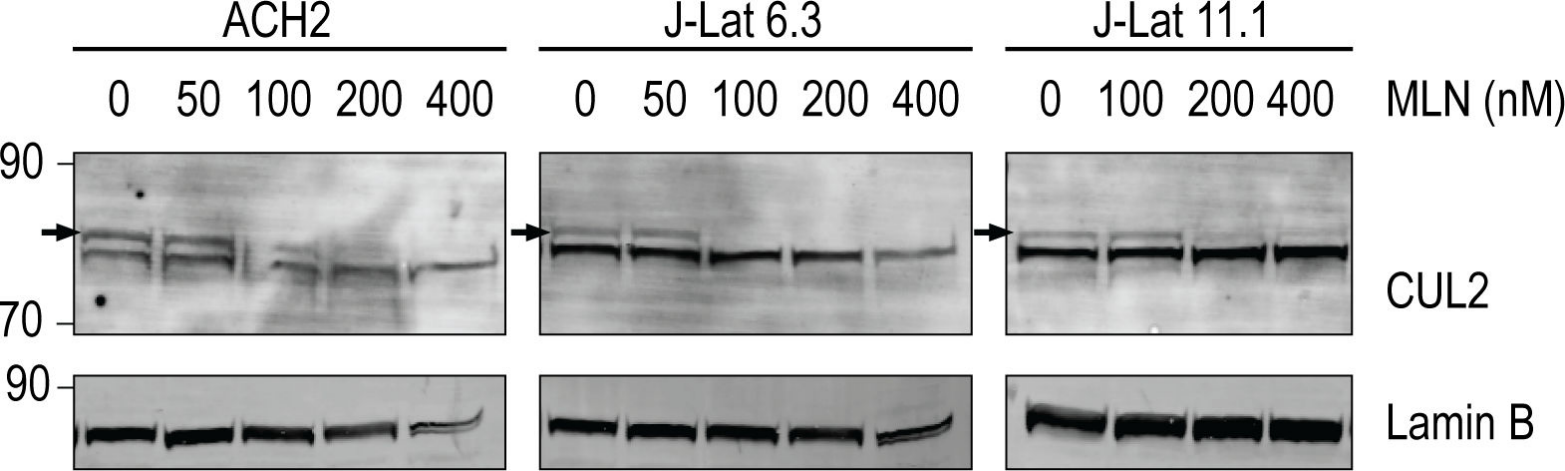
Cell lysates from ACH2, J-Lat 6.3, and J-Lat 11.1 cells were treated with increasing concentrations of MLN for 48 h. 0 refers to dimethyl sulfoxide control. The arrow indicates the neddylated form of CUL2 (30). Lamin B was used as the loading control.

We first studied the impact of inhibiting neddylation on the effects of LRAs in two well-characterized HIV provirus-containing T-cell lines: ACH2 and J-Lat cells. ACH2 cells contain a replication-competent provirus, while both the J-Lat 6.3 and 11.1 clones studied here harbor a replication-incompetent provirus encoding a GFP reporter. It is important to note that ACH2 cells have a single-nucleotide mutation in the trans-activation response (TAR) region of the HIV long-terminal repeat (LTR). Nevertheless, they maintain functional Tat and responsiveness to TNFα (27). Because MLN caused cell death of cancer cells *ex vivo* at concentrations from 500 nM to 3 µM (23, 28, 29), we first studied whether lower MLN concentrations inhibited Cullin neddylation in these HIV provirus-containing cell lines. Cullin 2 (CUL2) neddylation was inhibited at 100 nM in J-Lat 6.3 cells and at 200 nM in both ACH2 and J-Lat 11.1 cells (Fig. 1). We therefore used 200 nM MLN for all experiments shown here.

**Fig 2:**
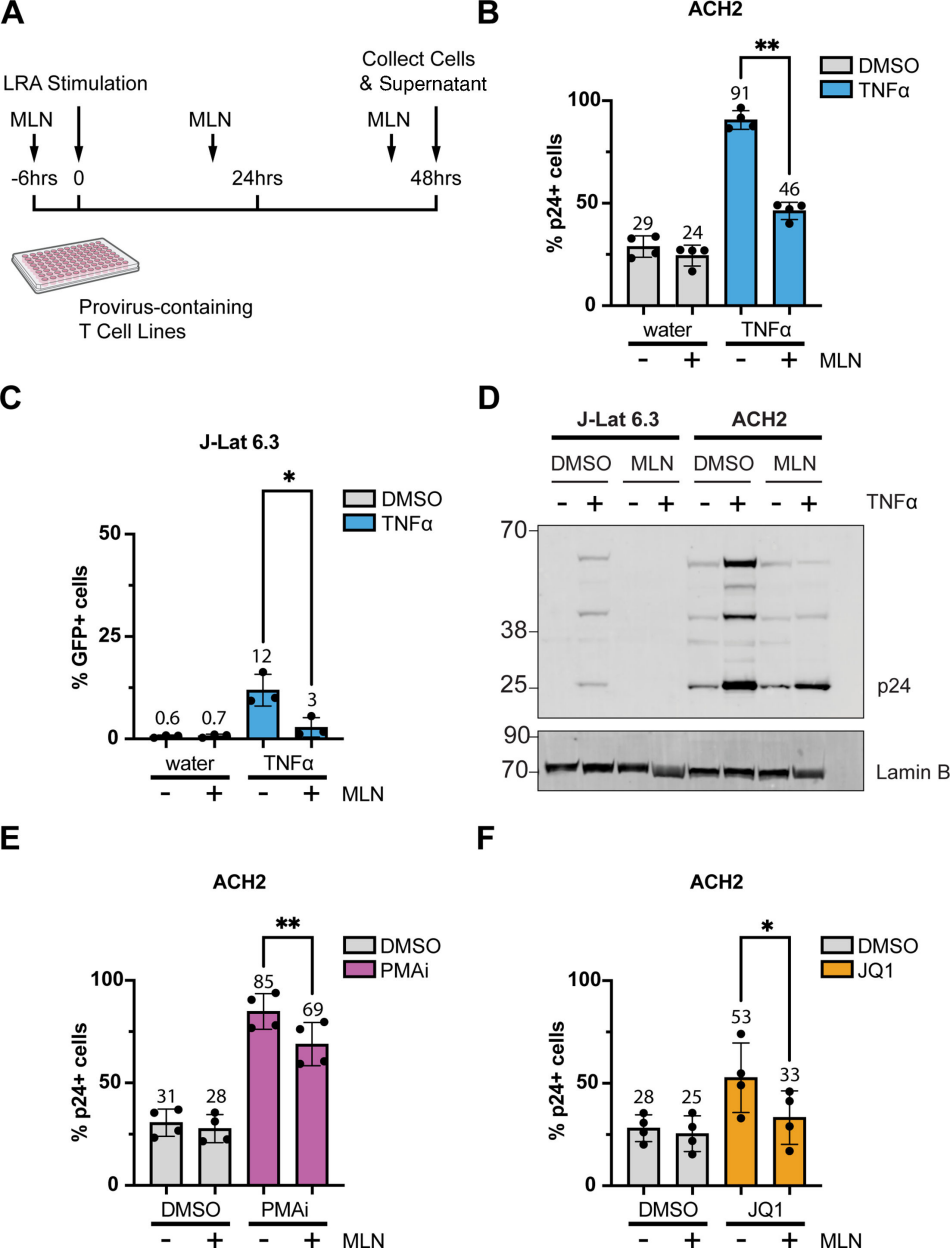
Intracellular HIV p24 expression after LRA treatment of ACH2 and J-Lat 6.3 cells was reduced with MLN. (**A**) Experimental design used to induce LRA-driven HIV in ACH2 and J-Lat 6.3 cells, with and without MLN exposure. (**B**) ACH2 cells were treated with 10 ng/mL TNFα (blue bars) or water (gray bars, control for TNFα) after 6 h pretreatment with dimethyl sulfoxide (DMSO; control for MLN) or 200 nM MLN as in (**A**). The percentage of HIV p24+ cells was measured via flow cytometry 48 h after TNFα treatment. (**C**) J-Lat 6.3 cells were treated, and the percentage of GFP+ cells was analyzed as in (**B**) with TNFα as the LRA. (**D**) Immunoblot of J-Lat 6.3 and ACH2 cell lysates, treated as in A). (**C, E**) ACH2 cells treated as in (**B**) with PMAi as the LRA. (**F**) ACH2 cells treated as in (**B**) with 100 nM JQ1 as the LRA. (**B and C, E and F**) Each dot represents one biological replicate (*n* = 4 in B, E, F, *n* = 3 in C), done in technical triplicate. Error bars show the standard deviation of biological replicates. Repeated-measures ANOVA was used for the indicated statistical comparisons, with adjustment for multiple comparisons using Turkey’s test: * *P* < 0.05, ** *P* < 0.005.

We started by testing the hypothesis that MLN would decrease the number of cells expressing HIV after LRA stimulation. After pretreatment with 200 nM of MLN, cells were stimulated with TNFα, PMAi, or JQ1 in the continued presence of MLN for an additional 48 hours (Fig. 2A). The percentage of cells expressing intracellular HIV-1 capsid protein, p24, was measured by flow cytometry 48 hours after adding each LRA. In ACH2 cells, MLN reduced the percentage of p24+ cells from 91% with TNFα alone to 46% with MLN plus TNFα (Fig. 2B; *P* = 0.001). Similarly, in J-Lat 6.3 cells, MLN reduced the percentage of GFP+ cells from 12% following TNFα alone to 3% with both (Fig. 2C; *P* = 0.03). Immunoblots of ACH2 and J-Lat 6.3 cell lysates also confirmed decreased levels of TNFα-induced intracellular p24 protein with MLN in both cell lines (Fig. 2D).

This phenotype was also seen with the other two LRAs, PMAi and JQ1, using the same experimental workflow. After PMAi treatment, MLN reduced the percentage of p24+ ACH2 cells from 85% with PMAi alone to 69% with PMAi and MLN (Fig. 2E; *P* = 0.003). After JQ1 treatment, the percentage of p24+ ACH2 cells was decreased from 53% to 33% by adding MLN (Fig. 2F; *P* = 0.04). This trend was consistent across additional experiments, not shown here, using various other J-Lat clones (e.g., 11.1 and 5a8) stimulated with either TNFα or PMAi. Overall, we observed that inhibiting neddylation with MLN reduced the percentage of cells expressing intracellular p24 or GFP after LRA stimulation across multiple cell lines and following three different LRAs. This indicates that a reduction in provirus reactivation when neddylation is inhibited is not specific to a single cell line, integration site, or LRA.

**Fig 3:**
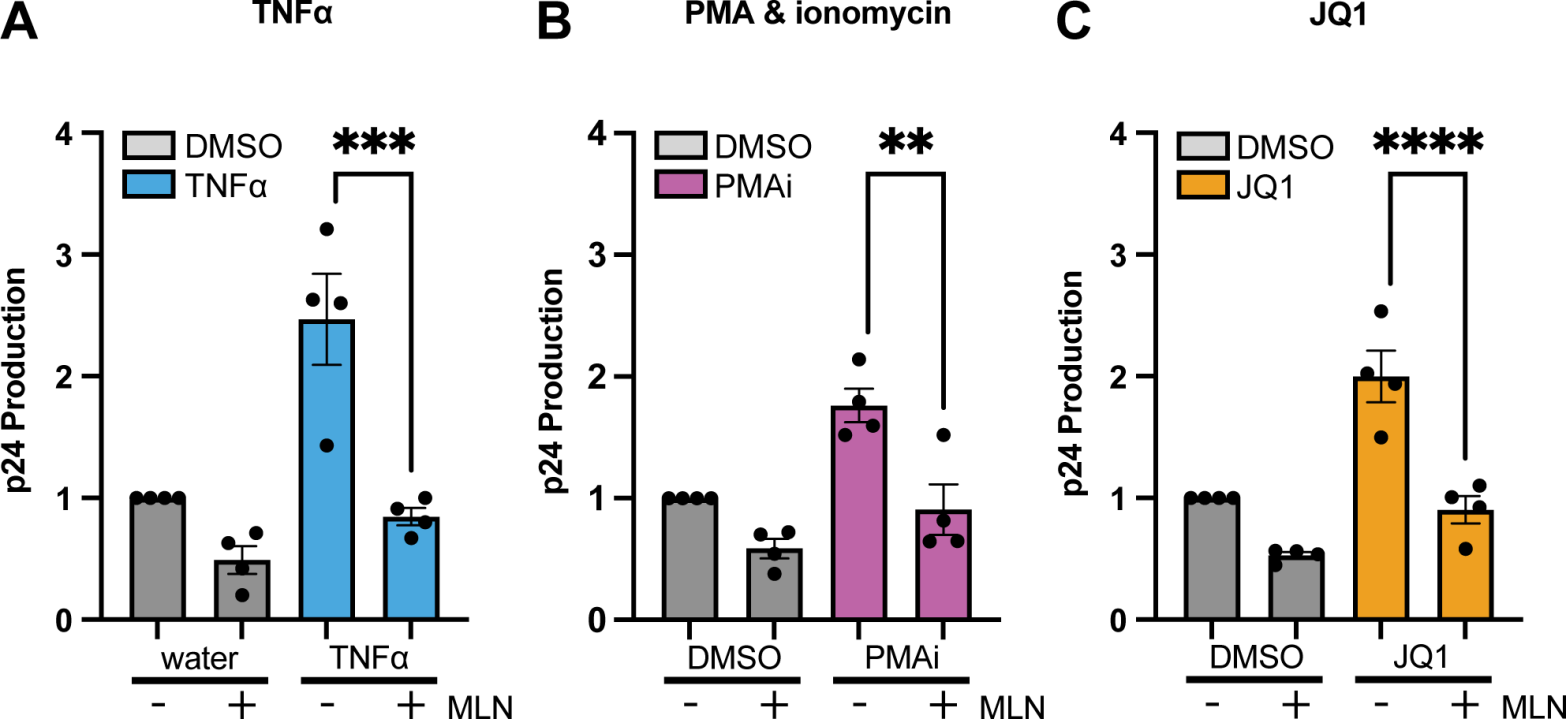
Levels of supernatant HIV p24 antigen after LRA treatment of ACH2 cells were decreased by MLN. ACH2 cells were pretreated for 6 h with dimethyl sulfoxide (DMSO) or 200 nM MLN prior to 48 h stimulation with either control or LRA, using these LRAs: (**A**) 10 ng/mL TNFα; (**B**) PMAi; and (**C**) 100 nM JQ1. Supernatant was collected 48 h after LRA treatment started. The Y-axis represents fold change in supernatant p24 concentration. Each dot represents one biological replicate completed in technical triplicate. Error bars show the standard deviation of four biological replicates. ANOVA was used for indicated statistical comparisons, with adjustment for multiple comparisons using Turkey’s test: ** *P* < 0.005, *** *P* < 0.0005, **** *P* < 0.0001.

Next, the amount of extracellular p24 in the cell culture supernatant of ACH2 cells was quantified using an HIV p24 ELISA to assess how much virus was produced from these cells (Fig. 3). Because J-Lat cells do not produce replication-competent virus, this and subsequent experiments assessing virus production and infectivity focused only on ACH2 cells. MLN significantly reduced the amount of extracellular p24 produced from ACH2 cells treated with TNFα (*P* = 0.0004), PMAi (*P* = 0.0025), or JQ1 (*P* < 0.0001), compared to the amount produced after each LRA alone (Fig. 3A through C). Together, these initial experiments showed that inhibiting neddylation reduced the number of ACH2 or J-Lat cells newly expressing HIV after these LRAs and also reduced the amount of virus released from ACH2 cells.

### Inhibiting neddylation reduced HIV *gag* RNA

**Fig 4:**
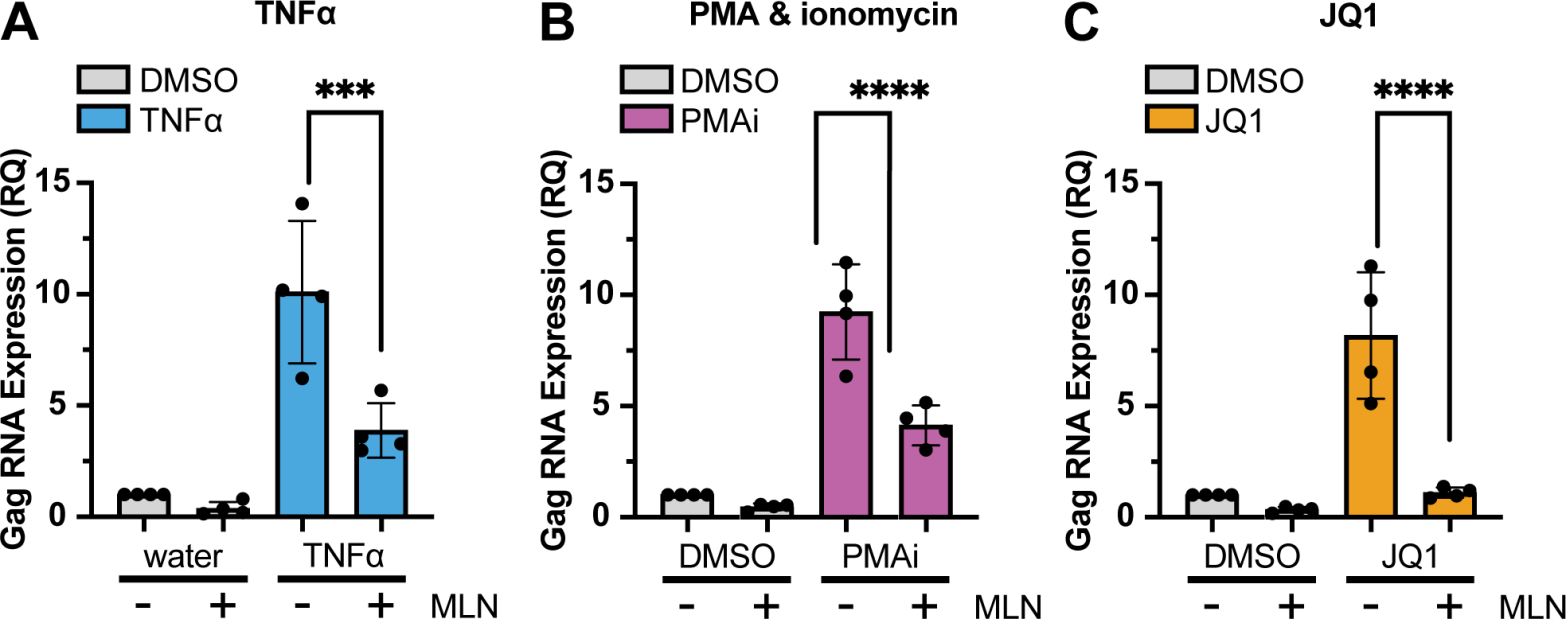
HIV *gag* RNA expression was decreased by MLN in ACH2 cells pretreated for 6 h with dimethyl sulfoxide (DMSO) or 200 nM MLN prior to 48 h of treatment with these LRAs: (**A**) 10 ng/mL TNFα; (**B**) PMAi; or (C) 100 nM JQ1. (**A–C**) RNA was extracted from cells 48 h after LRA treatment. The Y-axis represents relative quantification (RQ) of gag RNA. Each dot represents one biological replicate completed in technical triplicate. Error bars show the standard deviation of four biological replicates. ANOVA was used for indicated statistical comparisons: ***P < 0.0005, ****P < 0.0001, *n* = 4.

To explore the mechanism underlying reduced p24 expression and release after treatment with the three LRAs studied here, we assessed the impact of inhibiting neddylation via MLN on provirus transcription. We measured HIV *gag* gene expression in ACH2 cells following each LRA, with and without MLN. In ACH2 cells treated with TNFα and MLN, MLN reduced TNFα-stimulated *gag* RNA expression sixfold (*P* = 0.0003) compared to TNFα alone (Fig. 4A). MLN reduced *gag* RNA expression by fivefold (*P* < 0.0001) with PMAi treatment (Fig. 4B), and by sevenfold (*P* < 0.0001) with JQ1 treatment (Fig. 4C).

**Fig 5:**
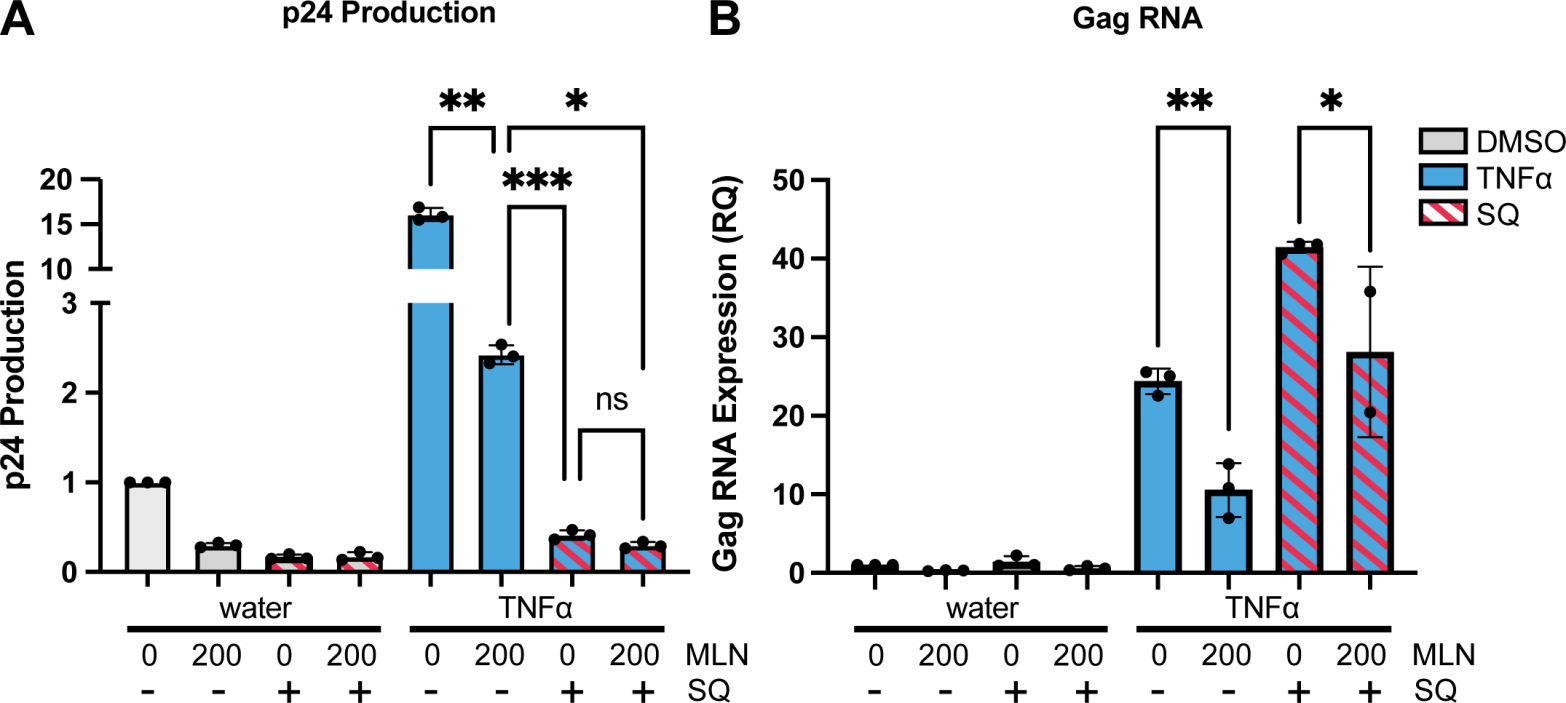
MLN reduced ACH2 extracellular p24 and intracellular *gag* RNA in the presence of saquinavir. ACH2 cells were treated as in Fig. 2A with 200 nM of MLN and 10 ng/mL TNFα (blue bars). 5 µM saquinavir (red stripes) was added at 0 h along with MLN. (**A**) Extracellular p24 was measured via ELISA. Supernatant was collected 48 h after LRA treatment. The Y-axis represents fold change in p24 concentration normalized to the dimethyl sulfoxide (DMSO) control. (**B**) Intracellular *gag* RNA expression. RNA was extracted from cells 48 h after LRA treatment. The Y-axis represents the fold change in *gag* RNA expression. (**A and B**) SQ = Saquinavir. Each dot represents one biological replicate completed in technical triplicate. Error bars show the standard deviation of three biological replicates. ANOVA was used for indicated statistical comparisons: *P < 0.05, **P < 0.005, ***P < 0.0005.

To determine whether effects on subsequent rounds of infection (e.g., superinfection) contributed to the reduction in *gag* RNA by MLN (Fig. 4), we cultured ACH2 cells in the presence and absence of saquinavir (Fig. 5). This HIV protease inhibitor specifically blocks cleavage of the Gag polyprotein, which is necessary for the maturation of newly released virions into infectious virions. Extracellular p24 decreased in the presence of saquinavir, whether MLN was also present or not, indicating that saquinavir effectively blocked processing of Gag polyprotein into p24 in both conditions (Fig. 5A). In the presence of saquinavir, MLN still reduced TNFα-stimulated HIV *gag* RNA (Fig. 5B). Thus, MLN reduced HIV *gag* RNA expression in cells stimulated by TNFα even when spreading infection was blocked by saquinavir. Overall, these data show that inhibiting neddylation hinders the induction of HIV *gag* gene expression by these three LRAs in ACH2 provirus-containing T cells.

### Inhibiting neddylation reduced HIV RNA by decreasing transcriptional initiation following TNFα, PMAi, or JQ1

Next, we tested whether the initiation or elongation of HIV transcription was responsible for this reduction in HIV RNA by quantifying transcripts associated with these steps of HIV transcription. Transcription of full-length HIV RNA by RNA polymerase II begins at the 5′ LTR promoter and pauses at the TAR stem-loop region in what is known as promoter proximal pausing (31–33). Blocks in elongation can cause RNA polymerase II not to extend beyond the TAR region, preventing elongation of the remaining transcript. Thus, the level of TAR transcripts serves as a measure of HIV transcriptional initiation, whereas the level of transcripts that include the region just downstream of TAR (“Long LTR”) quantifies elongation past the TAR region (34, 35). Prior literature has established that a greater than twofold excess of TAR over Long LTR transcripts in cells treated with both an LRA and MLN would signify that MLN inhibits HIV transcriptional elongation. Reduced TAR RNA levels with MLN, relative to LRA alone, would indicate that MLN decreased initiation of HIV transcripts (31, 34, 35).

**Fig 6:**
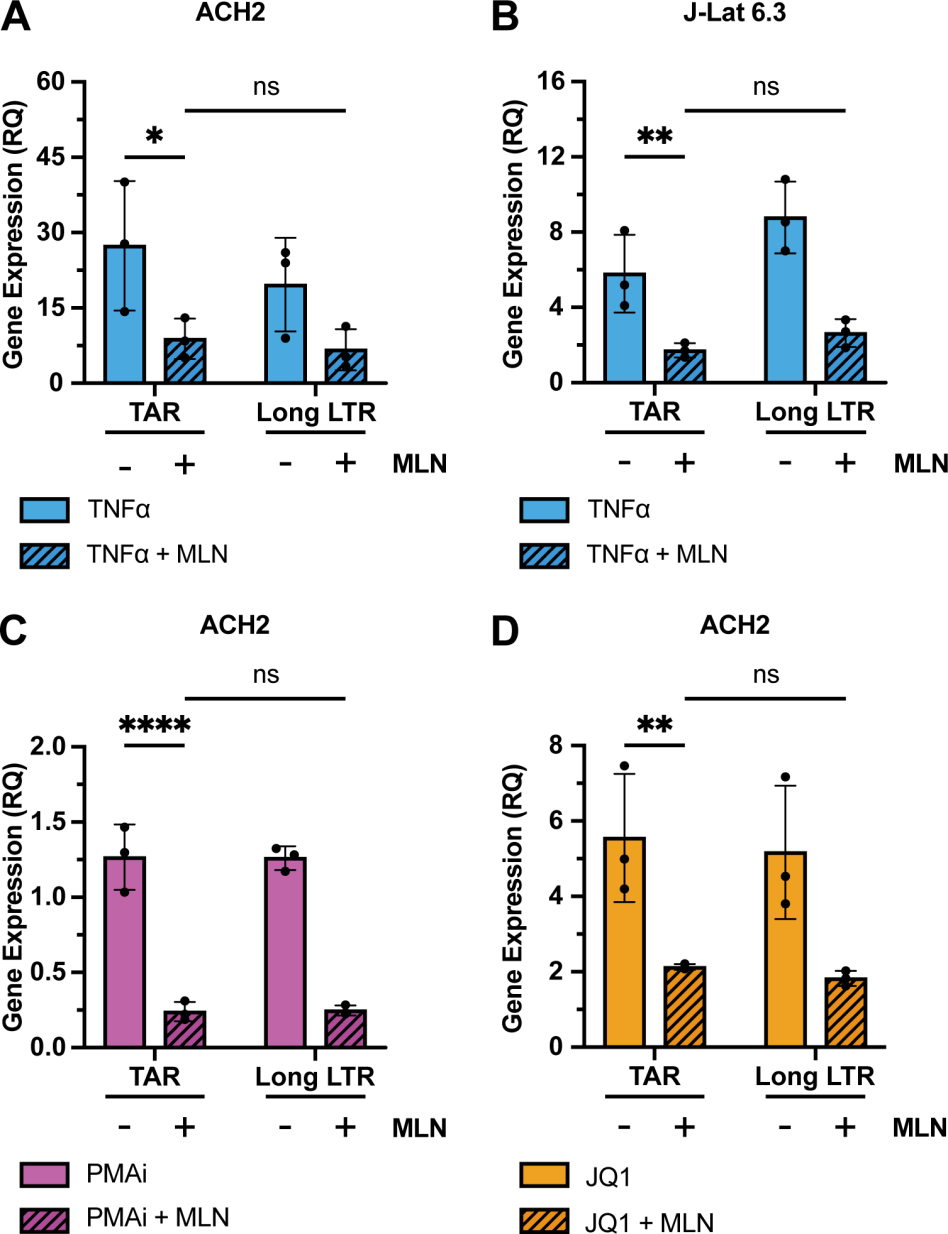
TAR and Long LTR transcripts were quantified in ACH2 (**A, C, and D**) and J-Lat 6.3 (**B**) cells after pretreatment for 6 h with dimethyl sulfoxide (DMSO) or 200 nM MLN prior to LRAs. MLN reduced TAR RNA levels after these LRAs: (**A and B**) 10 ng/mL TNFα; (**C**) PMAi; or (**D**) 100 nM JQ1. RNA was extracted 48 h after LRA treatment started. Gene expression was normalized to a DMSO control. Error bars show the standard deviation of three biological replicates completed in technical triplicate. ANOVA was used for indicated statistical comparisons: *P < 0.05, **P < 0.005, ***P < 0.0005, ****P < 0.0001.

With MLN treatment, TAR and Long LTR transcript levels were comparable to each other, with no statistically significant difference between the two transcripts in both ACH2 and J-Lat 6.3 cells treated with LRAs plus MLN (Fig. 6A through D). This indicates that there was no block to HIV transcript elongation with MLN. Conversely, when cells were treated with TNFα, the level of TAR RNA was significantly reduced with MLN compared to TNFα alone (*P* = 0.008 for ACH2, *P* = 0.004 for J-Lat 6.3), consistent with a block in initiation when neddylation is inhibited (Fig. 6A and B). This was also true in ACH2 cells treated with PMAi (*P* < 0.001) or JQ1 (*P* = 0.002) plus MLN, compared to the LRA-only control (Fig. 6C and D).

### Impact of neddylation on inhibitor of kB-alpha (IkBα) and LRA-driven HIV transcription

**Fig 7:**
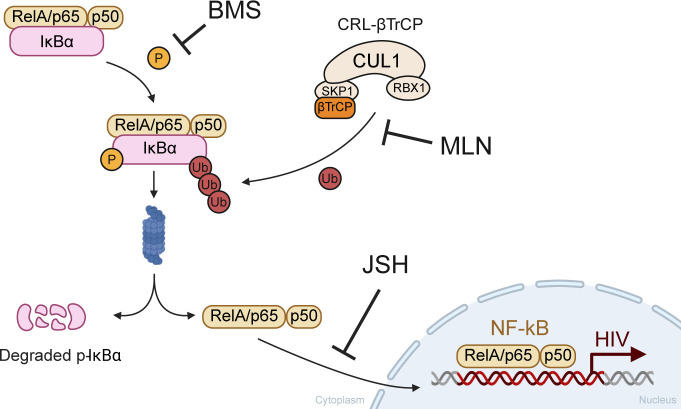
NF-kB is sequestered in the cytoplasm by phosphorylated IkBα (p-IkBα). Once IkBα is phosphorylated, it can be ubiquitinated by CRL^βTrCP^, which tags p-IkBα for proteasomal degradation. Degradation of p-IkBα releases NF-kB subunits RelA/p65 and p50 for translocation to the nucleus, where NF-kB can initiate HIV transcription at the HIV LTR. MLN inhibits p-IkBα degradation by inactivating CUL1 in CRL^βTrCP^ (20, 40). BMS inhibits phosphorylation of IkBα by IKK (41). JSH inhibits RelA/p65 translocation (42). Figure created on BioRender.

**Fig 8:**
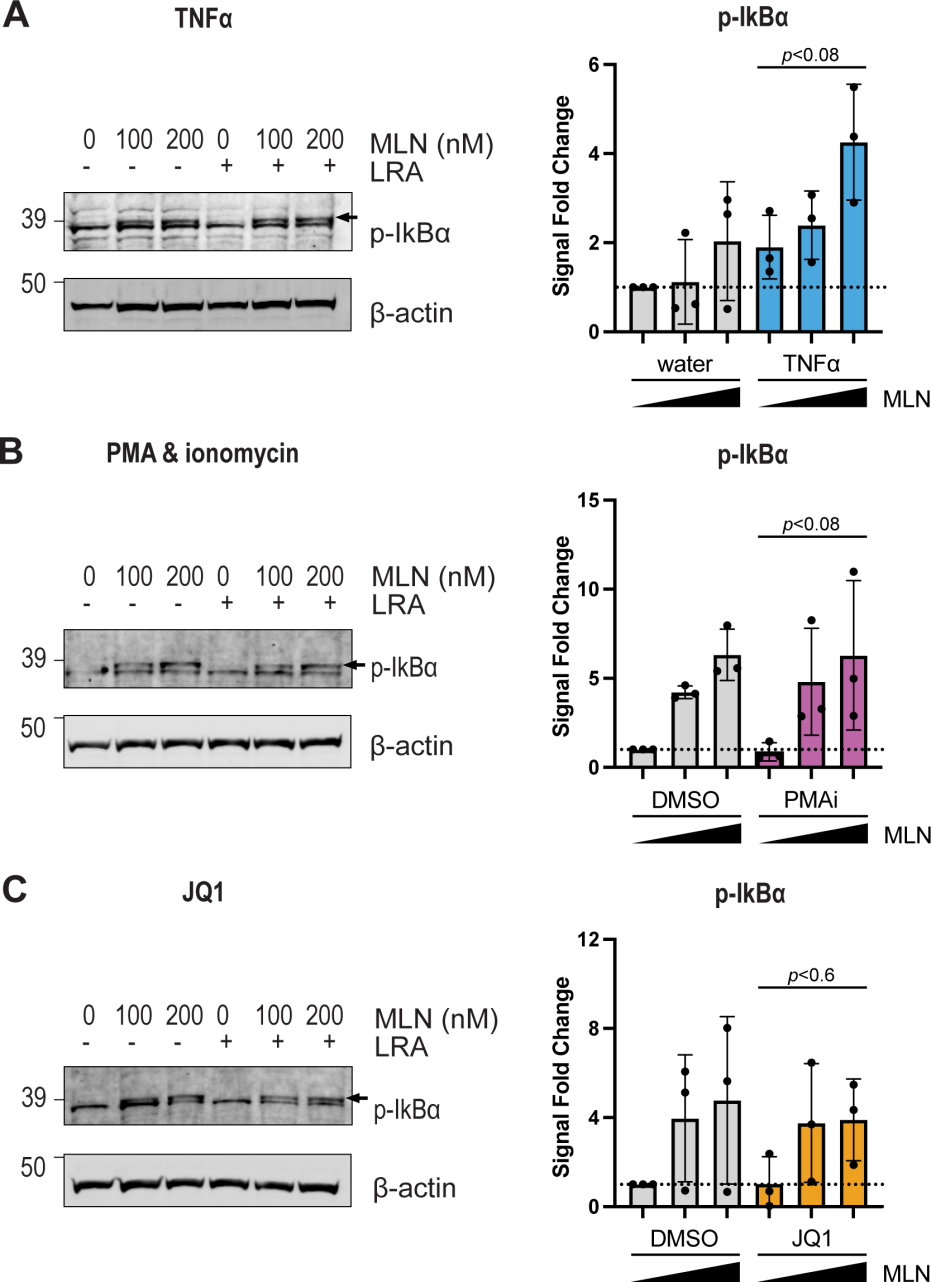
p-IkBα levels were assessed by immunoblot in ACH2 cells 24 h after control or LRA treatment. (**A**) ACH2 cells pretreated for 6 h with dimethyl sulfoxide (DMSO), 100, or 200 nM MLN, followed by 10 ng/mL TNFα. (**B**) ACH2 cells treated as in (**A**) with PMAi as the LRA. (**C**) ACH2 cells treated as in A with 100 nM JQ1 as the LRA. (**A–C**) Arrows point to p-IkBα. β-actin was used as a loading control. Quantification of p-IkBα signal is normalized to the loading control and the no treatment (DMSO only) control. The increase in p-IkBα was not statistically significant for TNFα (*P* < 0.08), PMAi (*P* < 0.08), or JQ1 (*P* < 0.6).

The results above suggested that these three LRAs share a common mechanistic pathway, at least in part, whose stimulation of HIV transcription was diminished by neddylation inhibition. We tested whether CRL-mediated degradation of a negative regulator of the canonical NF-kB pathway was impacted by MLN. In the canonical pathway, NF-kB is sequestered by inhibitor of kB-alpha (IkBα) in the cytoplasm (36, 37). When IkBα is phosphorylated by IkB kinase (IKK), the resulting p-IkBα must be ubiquitinated by a neddylation-activated CUL1 ubiquitin ligase (CRL^β-TrCP^), directing it to the proteasome (Fig. 7). After degradation of p-IkBα, NF-kB (RelA and p50) can then translocate to the nucleus to initiate transcription from the HIV LTR (6, 38, 39) (Fig. 7). Therefore, we tested the hypothesis that inhibiting neddylation would increase p-IkBα levels in ACH2 cells. Levels of p-IkBα were increased by 200 nM MLN, relative to the LRA-only control, after stimulation with TNFα (*P* < 0.08), PMAi (*P* < 0.08), or JQ1 (*P* < 0.6) (Fig. 8A through C). Given these suggestive results, two independent approaches were then taken to assess whether MLN impacted LRA-stimulated HIV transcription via the NF-kB pathway.

**Fig 9:**
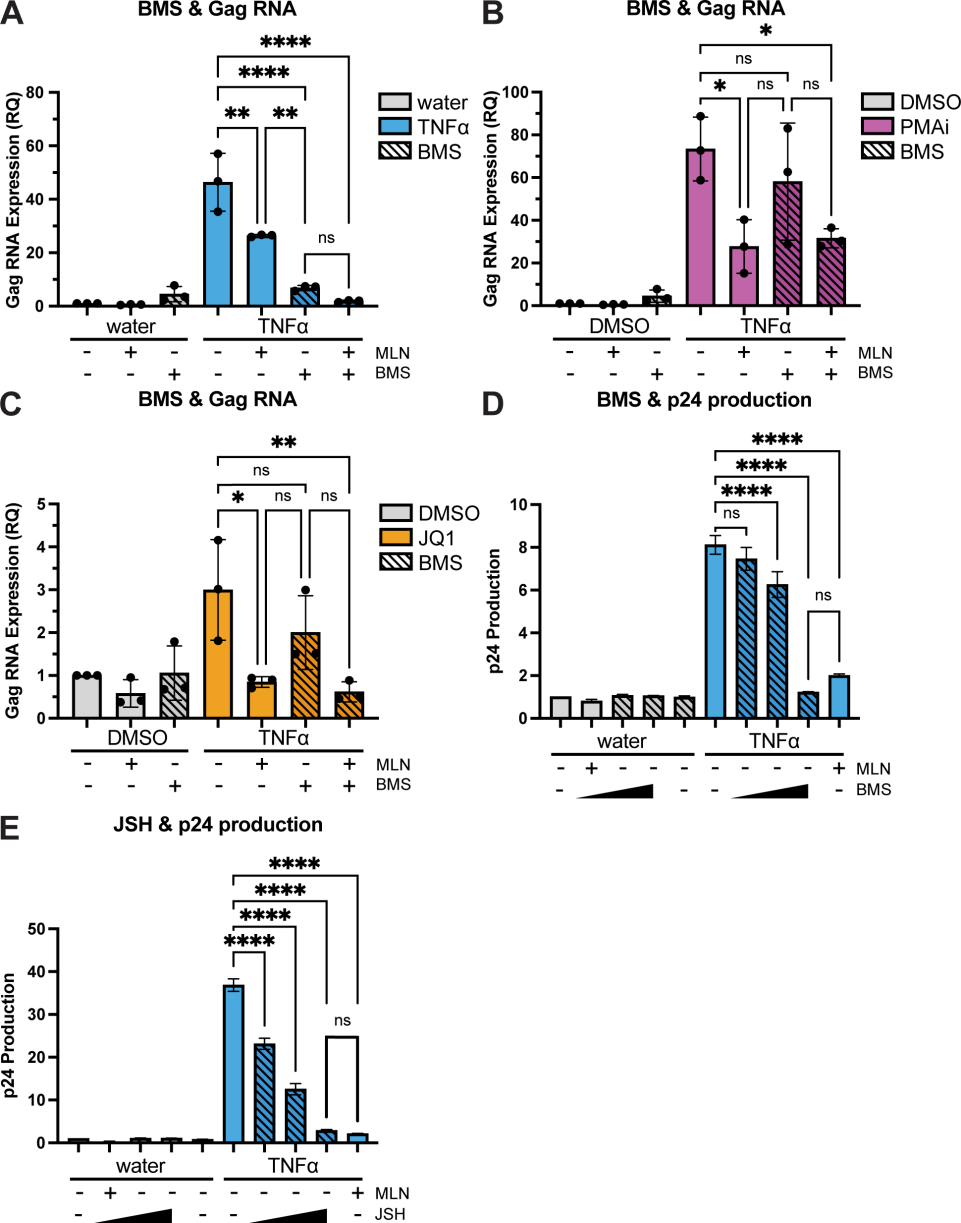
NF-kB inhibitors BMS or JSH mimic the effect of MLN on LRA-stimulated intracellular HIV RNA or extracellular p24 production. (**A–C**) Intracellular *gag* RNA expression in ACH2 cells pre-treated for 6 hours with control or 200 nM MLN prior to LRAs. BMS or dimethyl sulfoxide (DMSO) control was added at time 0. RNA was measured 48 h after LRA treatment. (**A**) 10 ng/mL TNFα, (**B**) PMAi, or (C) JQ1. Gene expression was normalized to the no-treatment control. The Y-axis represents relative quantification (RQ) of *gag* RNA. Error bars show the standard deviation of three biological replicates completed in technical triplicate. (**D and E**) Fold change in extracellular p24 was measured 48 h after TNFα treatment in combination with one of two different NF-kB inhibitors: (**D**) 0, 0.5, 1, and 5 µM BMS, added at 0 h, or (**E**) 0, 5, 10, or 50 µM JSH, added at 0 h. Error bars reflect the standard deviation of technical replicates. (**A–E**) ANOVA was used for indicated statistical comparisons: *P < 0.05, **P < 0.005, ****P < 0.0001.

A well-characterized, highly selective IKK inhibitor, BMS-345541 (BMS), was used here at 5 µM to inhibit the phosphorylation of IkBα, which is necessary for CRL-mediated degradation of p-IkBα (IKK-1 IC_50_ = 4 µM, IKK-2 IC_50_ = 0.03 µM) (41). 5 µM BMS significantly reduced TNFα-induced HIV *gag* RNA, relative to control (Fig. 9A; *P* < 0.0001). HIV RNA induced by either PMAi or JQ1 was also reduced by BMS as a single agent, albeit to a lesser degree that did not reach statistical significance (Fig. 9B and C). Of note, TNFα is documented to act on HIV transcription primarily, if not entirely, through NF-kB (27, 43). By contrast, PMAi and JQ1 are reported to stimulate HIV transcription via additional mechanisms as well as via NF-kB, as detailed in the discussion (44–47). In the presence of BMS, HIV *gag* RNA stimulated by each of these 3 LRAs was not significantly further reduced by adding MLN to the LRA and BMS (Fig. 9A through C). That is to say, BMS inhibition of IKK, which blocks formation of p-IkBα, precluded a further reduction in LRA-stimulated HIV transcription by MLN. No significant difference was seen in supernatant p24 levels between 5 µM BMS alone versus 200 nM MLN alone after TNFα stimulation (Fig. 9D).

A different inhibitor of the NF-kB pathway, which acts downstream of BMS to prevent nuclear translocation of RelA/p65 released by IkBα degradation, JSH-23 (JSH), was also studied (42). Extracellular HIV p24 was also similarly reduced by sole treatment with either 200 nM MLN or 50 µM JSH (Fig. 9E). Taken together, these results support the hypothesis that MLN acted via inhibition of the NF-kB pathway to reduce HIV transcription and subsequent p24 production.

### Inhibiting neddylation in CD4 T cells from ART-suppressed PLWH reduced HIV multiply spliced RNA

**Fig 10:**
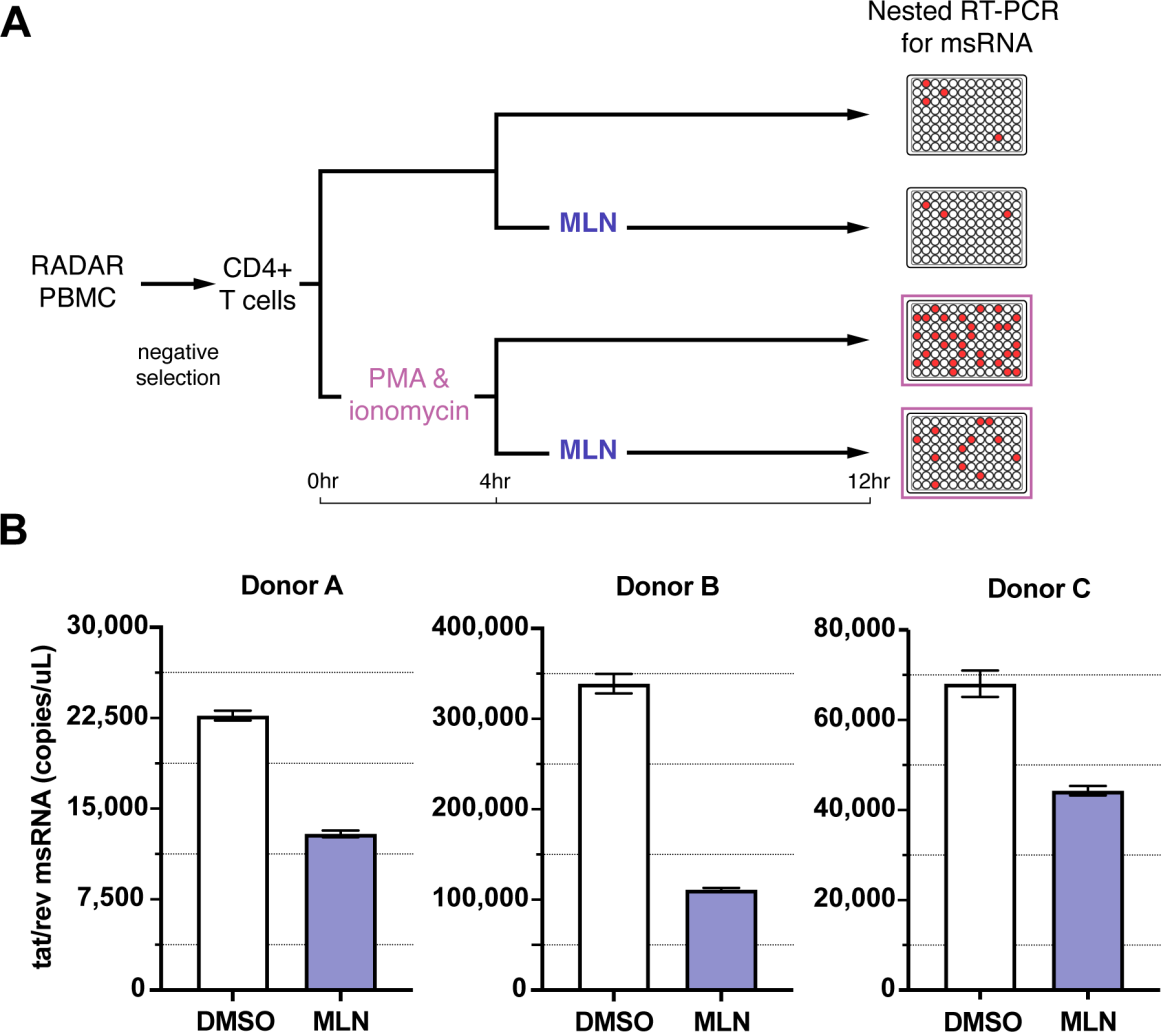
MLN decreased reactivation of provirus transcription by PMA and ionomycin from CD4+ T cells from ART-suppressed PLWH. (**A**) Experimental setup of the TILDA assay, as described in reference 51. CD4+ T cells were treated with PMAi or DMSO for 12 h total. After 4 h, either 200 nM MLN or DMSO was added to the cells for the remaining 8 h. A nested digital PCR platform was used to amplify *tat/rev* msRNA from each well, and the Applied Biosystems QuantStudio Absolute Q Digital PCR System was used to quantify RNA copies/μL. (**B**) msRNA copies/μL from CD4+ T cells after PMAi stimulation (pink in panel A) followed by either DMSO (white bars) or MLN (purple bars). Error bars show a 95% confidence interval.

We next expanded upon the results we found using cell lines by assessing PMAi-driven HIV provirus transcription in primary CD4+ T cells from ART-suppressed PLWH. Due to the low frequency of provirus-infected cells in the blood of ART-suppressed PLWH (48, 49), we used the highly sensitive Tat/rev Induced Limiting Dilution Assay (TILDA) to quantify PMAi-driven transcription *ex vivo* in cells from three donors (50, 51). After negative selection of CD4+ T cells from cryopreserved peripheral blood mononuclear cells (PBMCs), CD4+ T cells were stimulated for a total of 12 hours with PMAi as is standard in this assay (50, 51). Cells were treated with dimethyl sulfoxide (DMSO) or 200 nM of MLN starting 4 hours after PMAi stimulation. After 12 hours, cells were collected and multiply spliced tat/rev RNA (msRNA) was measured using a plate-based digital PCR platform (Fig. 10A).

MLN treatment reduced msRNA after PMAi stimulation of cells from all three donors (Fig. 10B). PMAi-driven provirus transcription was reduced with MLN by 35%, 67%, and 43%, respectively, in each donor’s cells compared to DMSO control. As expected, variation in HIV reservoir size across individuals resulted in differing magnitudes of msRNA between donors. More information on each donor is found in Table 1. In summary, inhibiting neddylation limited increases in provirus transcription after PMAi stimulation in this primary cell model, supporting the relevance of results in cell lines.

**TABLE 1:** Information on cells studied in Fig. 10^*a*^

	Donor A	Donor B	Donor C
Age (years)	24	35	33
Sex assigned at birth	Male	Male	Male
Gender identity	Man	Man	Man
Viral load	Undetectable	Undetectable	Undetectable
CD3/CD4 (%)	32	ND	40
CD3/CD4 (absolute count)	611	ND	805
ART medication regimen	Elvitegravir/cobicistat/emtricitabine/tenofovir fixed-dose combo (Stribild)	Abacavir/dolutegravir/lamivudine fixed-dose combo (Triumeq)	Bictegravir/emtricitabine/tenofovir fixed-dose combo (Biktarvy)
Years since 1st started ART	2.1	6.7	3.6
Seroconverted during study	Yes	No	No

^*a*^ CD4+ T cells came from ART-suppressed, de-identified PLWH participating in the RADAR cohort (52). Undetectable plasma viral load was defined as <40 copies/mL. ND = not determined. Seroconverted refers to RADAR study participants who seroconverted during the RADAR study and were subsequently put on ART treatment.

### Inhibiting neddylation protected APOBEC3G from degradation in ACH2 cells and decreased infectivity of LRA-stimulated virions

We have shown thus far that inhibiting neddylation via MLN curtailed increases in the number of HIV expressing cells, virus production, HIV *gag* RNA, and HIV transcriptional initiation after LRA treatment in provirus-containing T-cell lines. MLN also reduced PMAi-driven provirus transcription in CD4+ T cells from ART-suppressed PLWH. Given that MLN has been demonstrated to reduce infectivity of virions in *de novo* HIV infections (18–20), we next studied the infectivity of viruses produced from LRA-stimulated ACH2 cells containing Vif-positive provirus.

**Fig 11:**
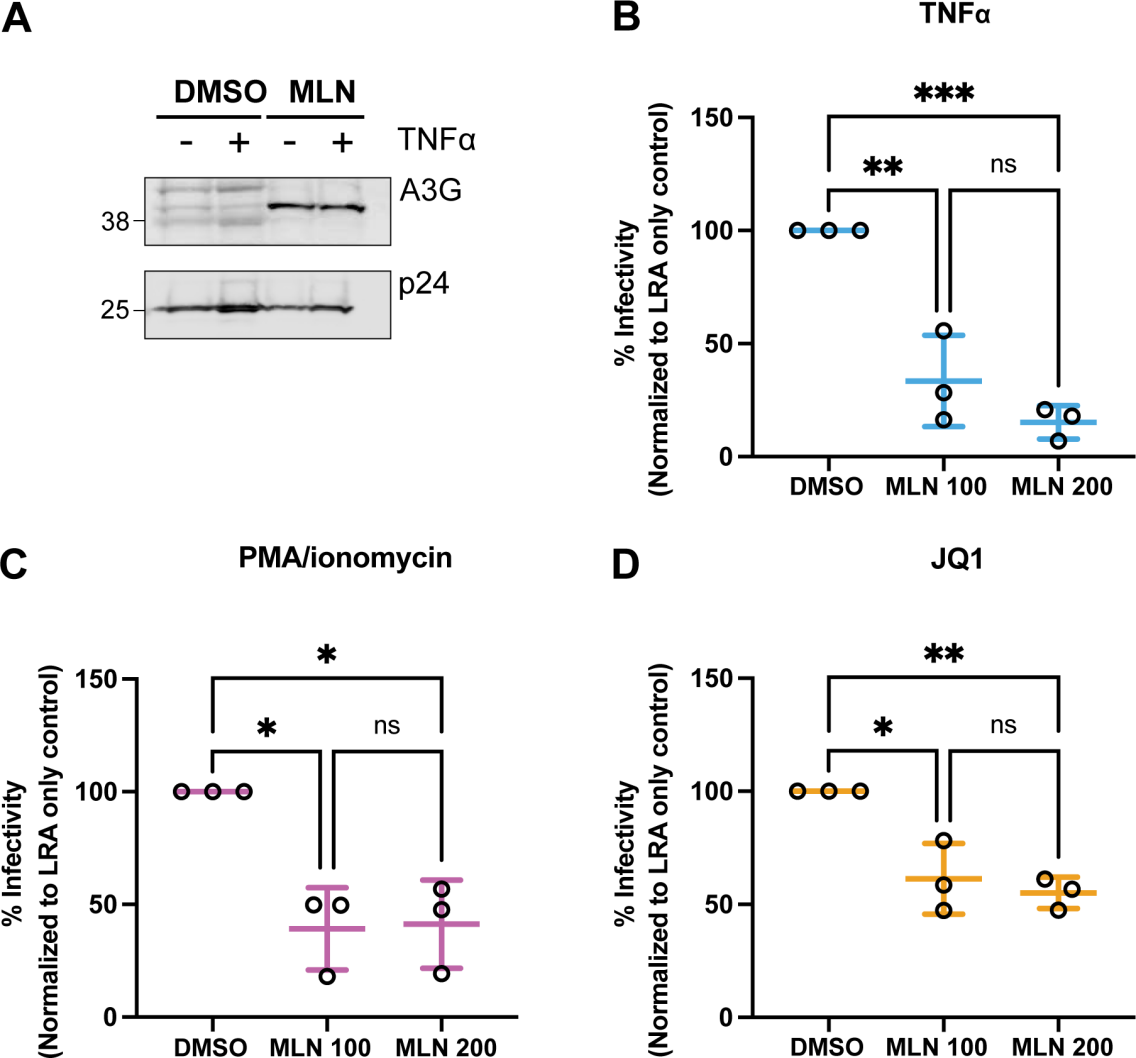
MLN increased virus incorporation of A3G and decreased infectivity of virions produced by ACH2 cells following LRAs. (**A**) HIV was pelleted from ACH2 cells culture supernatant that were pretreated with DMSO or 200 nM MLN followed by 48 h of 10 ng/mL TNFα (or control) as per Fig. 2A. Pelleted virions were lysed and run on an immunoblot that was probed for A3G and p24. One representative image is shown. (**B–D**) Percent infectivity of virions from supernatants of LRA-treated ACH2 cells pretreated with MLN or DMSO control. (**B**) 10 ng/mL TNFα; (**C**) PMAi; or (**D**) 100 nM JQ1. Cells were treated as per Fig. 2A. The LRA+ DMSO condition represents 100% infectivity. Error bars show the standard deviation of three biological replicates completed in technical triplicate. One-way ANOVA was used for indicated statistical comparisons: *P < 0.05, **P < 0.005, ***P < 0.0005.

 If A3G escapes degradation in HIV-producing cells and is packaged into virions, it potently reduces virion infectivity (53–55). We tested whether Vif-positive virions produced from ACH2 cells after LRA stimulation, in the presence of MLN that inhibits activation of CRL^Vif^, would be less infectious due to increased A3G incorporation (18, 19). We pelleted virus from the supernatant of ACH2 cells that were pretreated with MLN or DMSO prior to TNFα stimulation (as in Fig. 2A). MLN resulted in higher A3G incorporation in virions with and without TNFα treatment (Fig. 11A).

The infectivity of virions was studied by infecting TZM-bl cells with equal amounts of supernatant virus from ACH2 cells with and without MLN treatment and assessing the relative luminescence units (RLU) (56). Vif-positive virions produced from MLN and LRA-treated ACH2 cells had reduced virion infectivity compared to virions from LRA-treated cells without MLN exposure (Fig. 11B and D). Infectivity of virions from TNFα-treated ACH2 cells was reduced by 67% (*P* = 0.002) with 100 nM MLN and 85% (*P* < 0.0004) with 200 nM MLN (Fig. 11B). MLN reduced the infectivity of virions induced by PMAi by 61% (*P* = 0.007) with 100 nM MLN and 59% (*P* = 0.008) with 200 nM (Fig. 11C). With JQ1 treatment, MLN reduced infectivity by 39% (*P* = 0.007) with 100 nM MLN and 45% (*P* = 0.003) with 200 nM MLN (Fig. 11D). Taken together, MLN increased A3G incorporation into virions released from ACH2 cells after each of these three LRAs, decreasing infectivity. Thus, inhibiting neddylation also limited infection spread in provirus-containing cell cultures.

## DISCUSSION

This study identified that neddylation increased LRA-stimulated proviral HIV transcription by broadly inhibiting neddylation using MLN. With that same approach, neddylation was also found to decrease A3G incorporation into LRA-reactivated HIV virions. The *ex vivo* results will be summarized. Hypotheses suggested by this study that could be tested in future animal model research using MLN at the time ART is stopped, rather than with an LRA during ART, will be discussed. Rationale for future discovery of targeted alternatives to broadly acting MLN will also be described below.

First, MLN limited LRA-driven HIV transcription and virus production by TNFα, PMAi, and JQ1 in provirus-containing ACH2 and J-Lat 6.3 T-cell lines, diminishing the intended effects of an LRA (Fig. 2 to 4). MLN likely impacted the cells initially reactivating provirus because LRA-stimulated HIV RNA and p24 antigen production were each also decreased when saquinavir blocked HIV spread in ACH2 cultures (Fig. 5). MLN also decreased PMAi-induced transcription in CD4+ T cells from ART-suppressed PLWH (Fig. 10), indicating relevance to primary cells. Provirus transcription stimulated by each of these LRAs was decreased at the initiation step by MLN (Fig. 6), raising the hypothesis that the three LRAs share a common mechanism by which neddylation up-regulates HIV transcription.

Several lines of evidence presented in this study support that a common mechanism among the three LRAs was an MLN-mediated decrease in degradation of an inhibitor of NF-kB, p-IkBα (Fig. 7). p-IkBα levels were non-significantly increased by MLN (Fig. 8). BMS, which inhibits phosphorylation of IkBα by IKK (41), and JSH, which inhibits nuclear translocation of RelA/p65 (42), each individually diminished HIV transcription and precluded a further reduction in LRA-stimulated HIV transcription by adding MLN (Fig. 9). Additional support came from the differing magnitudes by which MLN decreased HIV expression stimulated by each of the three LRAs. MLN decreased the number of p24+ cells and extracellular p24 levels more when given with TNFα treatment than the other two LRAs (Fig. 9A through C). These results are consistent with prior reports that PMAi and JQ1 each act to increase HIV transcription via separate mechanisms in addition to NF-kB. PMA acts via NF-kB (44), while ionomycin increases NFAT signaling to further activate transcription at the HIV LTR (45). JQ1 has been previously reported to increase HIV transcriptional activation via several mechanisms: epigenetic effects, enhancement of pTEFb recruitment to TAR by HIV Tat, as well as indirectly increasing NF-kB transcription (46, 47, 57–61). Taken together, these results suggest that MLN is decreasing HIV expression after TNFα, PMAi, or JQ1 via inhibition of canonical NF-kB signaling. Determining whether NF-kB binding to the HIV LTR is decreased when neddylation is inhibited in the presence of each of these single LRAs, relative to the LRA alone, will help validate this hypothesis in the future. CRL^β-TrCP^ also degrades a protein with a similar function as p-IkBα in the non-canonical NF-kB pathway, p100 (62, 63). This suggests future study of MLN’s effects on non-canonical NF-kB signaling.

In addition to its effects on HIV transcription, broadly inhibiting neddylation with MLN was also found to block A3G degradation in LRA-stimulated ACH2 cells, reactivating Vif-positive virus production. MLN increased A3G packaging into virions and potently diminished infectivity of those virions (Fig. 11). This extended previous findings from previous studies of *de novo* HIV infections (18, 19). This is relevant because others have reported that CD4+ T cells harboring A3G-hypermutated, replication-defective proviruses caused by infection with virions containing increased A3G can more potently activate HIV-specific CD8+ cytolytic T cells (CTL) than do CD4+ T-cell proviruses lacking this magnitude of A3G hypermutation. This has been seen in both *ex vivo* and *in vivo* experiments (64, 65). Other effects reported in previous studies also impacted anti-HIV immunity. DNA repair mechanisms induced by A3G-hypermutated HIV genomes upregulated expression of a ligand on CD4 T cells that activates natural killer (NK) cells, specifically increasing NK cell-mediated killing of HIV-infected CD4+ T cells containing such A3G-hypermutated proviruses (66). Dendritic cells infected with lethally A3G-hypermutated HIV have also been reported to more effectively present HIV antigens to CD8+ CTL to enhance their specific recognition/cytolysis of those A3G-hypermutated HIV-expressing CD4+ T cells (67). Others also verified that antigens are expressed from defective HIV proviruses and recognized by CD8+ CTLs to shape the dynamic changes in the proviral landscape in CD4+ T cells *in vivo* over time (68).

Several new research directions are suggested by our results, including *ex vivo* studies if the broad effects of MLN also decrease Vpu-mediated degradation of tetherin and/or Vpr-mediated degradation of cellular factors impacting cell cycle, apoptosis, and/or transcription (69–72). The combined effects of MLN on both HIV transcription and A3G virion incorporation, and the HIV-specific immunity-enhancing effects of increased A3G, summarized above (64–68), suggest animal model research evaluating whether these effects can help control HIV rebound from latent reservoirs off-ART. A current paradigm in HIV cure research, often called “shock and kill,” adds LRA(s) to ART. An LRA aims to expose newly HIV-expressing cells to clearance by immune effector cells, while ART protects against reactivated HIV spread. However, administering LRA(s) during ART has not yet led to substantial reactivation of HIV antigen expression or detectable clearance of newly HIV-expressing cells by immunity. In contrast to adding an LRA to ART, stopping ART reliably leads to robustly rebounding viremia, reflecting a spreading infection. We hypothesize that MLN given when ART stops in an animal model of HIV infection may add to lessons learned here about leveraging mechanisms for a different paradigm than using LRA(s) during ART: attempting to use MLN to curtail HIV rebound from latency when ART stops. Studying MLN when ART stops in animal models can test whether and how uninfected CD4+ T cells may be protected from infection by increased levels of virion A3G and/or if CD4+ T or dendritic cells expressing A3G-hypermutated HIV genomes may be better recognized and eliminated by immune effector cells (CD8+ T and NK cells), as described previously (64–68). This hypothesized effect of A3G to increase anti-HIV immunity is supported by reports that adding an adjuvant that increases cellular A3G improved innate and adaptive immune responses to SIV vaccines in rhesus macaques (73–75). Such animal model research using MLN is envisioned as only a first step in developing such a new research paradigm, if it validates these hypotheses.

Although useful for characterizing the above mechanisms in animal models, MLN’s broad effects on inhibiting neddylation-activated ubiquitination of many cellular proteins (23–25, 40, 76) and its potential cytotoxicity on uninfected cells limit its potential for clinical application in HIV. A risk of cytotoxicity that is acceptable in those with cancer does not seem appropriate for ART-treated persons with HIV. Development of more specific, less cytotoxic inhibitors of one or a few CRLs is preferable and may become possible. New knowledge explicates how, specifically and notably, the NEDD8 modification of CUL1 promotes the transfer of ubiquitin to p-IkBα by CRL^β-TrCP^ (77), potentially enabling more specific targeting of that CRL to either increase or decrease its ubiquitinating activity. Molecular understanding of how neddylation activates CUL1 in CRL^β-TrCP^ may also inform targeting of some of the other eight Cullins, which scaffold regulation of many additional protein substrates. This, and efforts to improve broad neddylation inhibitors for cancer treatment (4), could lead to new compounds targeting specific CRLs and/or their targets. Lead compounds that can either increase or decrease the degradative capacities of specific CRLs by modulating neddylation or its effects on CRL ubiquitinating activity may emerge as candidates with more specificity than MLN and with less potential for uninfected cell cytotoxicity. We suggest that more specific agents than MLN be available for study before refining operational protocols for this proposed new approach for sustaining HIV remission that we hypothesize will enhance anti-HIV immunity when ART stops, first in animal models and then in any potential clinical research.

This hypothesis-generating study is also limited in not providing enough pre-clinical data with the lower concentration of MLN used here to yet enable advancing such concepts. Longer-term evaluations of MLN in both *ex vivo* and animal model studies of HIV infection will be needed to enable animal experiments. Another limitation is that the separation and study of different T-cell subtypes, or other immune cells implicated as HIV reservoirs, was not possible given the small number of blood cells available from ART-suppressed PLWH.

In summary, this work highlights neddylation as a targetable mechanism increasing HIV transcription. MLN reduced latency reversal, including in blood CD4+ T cells from ART-suppressed PLWH. Results suggest that MLN prevented degradation of p-IkBα to limit NF-kB-driven HIV transcription. MLN also increased reactivated virion A3G. This result, and earlier studies showing that increased virion A3G enhanced anti-HIV immunity (64–68), suggests testing whether using MLN when latent HIV spontaneously reactivates after stopping ART can help control HIV rebound off-ART. Studying this in an animal model of HIV infection can begin to validate whether specific proteins and pathways affected by MLN in this study may contribute to the development of a new research strategy to sustain HIV remission off-ART. While broad inhibition of neddylation is unlikely to be clinically applicable to HIV management, the discovery of an agent specifically impacting one or a few proteins identified by studying MLN, such as IkBa or A3G, may help advance such an approach.

## MATERIALS AND METHODS

### Cell culture and cell lines

Suspension cell lines were cultured in RPMI with L-glutamine (Corning) plus 10% fetal bovine serum (FBS), 50 IU/mL penicillin, and 50 μg/mL streptomycin (Corning, #30-002-CI) and maintained at 37°C and 5% CO_2_. ACH2 cells (ARP-349) and J-Lat 6.3 (ARP-9846) were obtained from BEI Resources (formerly the NIH HIV Reagent Program). J-Lat 11.1 cells, originally developed by Dr. Eric Verdin (78), were a generous gift from Dr. Steven Wolinsky at Northwestern University. TZM-bl cells (ARP-8129) were obtained from BEI Resources and cultured in DMEM (Corning) with 10% FBS, 50 IU/mL penicillin, and 50 μg/mL streptomycin as above.

### Reactivation of provirus-containing T-cell lines and LRAs

Provirus-containing T-cell lines (ACH2 or J-Lat) were cultured at 2 × 10^6^ cells/mL and pretreated for 6 hours with 100 or 200 nM of MLN (Cayman Chemical, #15217) or equivalent amounts of DMSO. MLN was replenished every 24 hours throughout the course of the experiment (three times total, including the initial pretreatment) (see Fig. 2A). After 6 hours of pretreatment, cells were stimulated with one of the following LRAs: 10 ng/mL TNFα (PeproTech, #300-01A), a 1:125,000 dilution of PMA and ionomycin (PMAi) (eBioscience Cell Stimulation Cocktail, #00-4970-93), or 100 nM JQ1 (Tocris, #4499). Cells treated with TNFα were cultured in RPMI with only 1% FBS (plus penicillin and streptomycin as above). Controls were treated with equivalent amounts of DMSO (PMA/i, JQ1) or water (TNFα). Cells and supernatants were collected for downstream assays 48 hours after LRA treatment, unless otherwise specified.

### Flow cytometry

All experiments were conducted on the BD Biosciences LRS Fortessa Analyzer. Cells were collected 48 hours after LRA stimulation and stained with either Ghost Dye Red 710 Viability Dye (Tonbo Bioscience, #13-0871-T100) or LIVE/DEAD Fixable Blue Dead Cell Stain Kit (Invitrogen, #L34962) to measure cell viability. ACH2 cells were fixed with 4% paraformaldehyde for 30 minutes, then permeabilized and stained with anti-p24 KC57-FITC (Beckman Coulter, #6604665). Analysis was performed by gating on live cells followed by gating on GFP+ cells (J-Lats) or p24+ cells (ACH2). The percentage of p24+ or GFP+ cells in each condition was plotted. Statistical analysis was done in Graphpad Prism. Conditions were compared using Repeated Measures (RM) one-way ANOVA with the Geisser-Greenhouse Correction. Turkey’s multiple comparisons test, with individual variances, was computed for each comparison.  

### Immunoblotting

Cell pellets containing the same number of cells across conditions were collected 48 hours after treatment with LRAs and were lysed in a 1% NP-40-based lysis buffer with protease inhibitor (Roche, #11836145001) and subsequently sonicated to lyse the nuclear membrane as we have previously described (79). Cells were centrifuged at 10,000 × *g* for 12 min at 4°C and denatured using 10% DTT (Sigma, D0632) and 4× Bolt LDS sample buffer (Thermo Fisher Scientific, #B0008).

To pellet viruses, culture supernatant was filtered through a 0.45 µm filter, layered over a 20% sucrose cushion, and ultracentrifuged at 32,000 rpm at 4°C for 1.5 hours (Beckman Coulter SW41Ti). Pellets were resuspended in buffer (20 µL Bolt LDS sample buffer, 8 µL DTT, and 12 µL lysis buffer) and transferred to Eppendorf tubes.

All samples were then boiled for 7 minutes at 92°C and underwent electrophoresis through a 4%–12% Bolt Bis-Tris gel (Invitrogen). Proteins were transferred to a PVDF membrane (Thermo Scientific, #88518) and probed with primary antibodies. The following antibodies were used: anti-CUL2 (Santa Cruz, #166506), anti-phospho-IkB alpha (Proteintech, #82349-1-RR), anti-p24 (183-H12–5C, Tennessee Center for AIDS Research Virology Core), anti-lamin B1 (Proteintech, #66095-1) or anti-lamin B1 (GeneTex, #GTX103292), anti-beta-actin (Proteintech, #66009), and anti-A3G C17 (ARP-10082, polyclonal anti-human APOBEC3G, obtained from BEI Resources, formerly NIH HIV Reagent Program). Fluorescently labeled secondary antibodies (Li-Cor #926-68071 and #926-32210) were used in conjunction with the Li-Cor Odyssey CLx imaging system to visualize membranes. Quantification of immunoblots was done using Li-Cor Empiria Studio 3.2. Quantification of bands was done by dividing the signal of the band of interest by the signal of the corresponding loading control. Values were then normalized to the DMSO/no treatment control.

### Gene expression analysis

Total RNA was isolated from cells using the Qiagen RNeasy Mini Kit (#74106) according to the manufacturer’s protocol. cDNA was immediately synthesized from extracted RNA using Invitrogen SuperScript III first-strand synthesis kit (#18080-051) according to the manufacturer’s instructions. Following cDNA synthesis, viral transcripts were assessed by RT-qPCR. For HIV *gag*, the following primers were used: F: 5′ TGCTATGTCAGTTCCCCTTGGTTC TCT 3′, R: 5′ AGTTGGAGGACATCAAGCAGCCATCGAAAT 3′. The cycling conditions were as follows: 95°C for 15 min followed by 40 cycles of 95°C for 15 s, 57°C for 30 s, and 72°C for 30 s. HIV TAR and Long LTR were assessed based on this previously described method (35). TAR was assessed using F: 5′ GTCTCTCTGGTTAGACCAG 3′, R: 5′ TGGGTTCCCTAGYTAGCC 3′, and Long LTR using F: 5′ GCCTCAATAAAGCTTG CCTTGA 3′, R: 5′ GGGCGCCACTGCTAGAGA 3′. The cycling conditions were 95°C for 5 min followed by 40 cycles of 95°C for 15 s, 60°C for 30 s, 72°C for 1 min. RT-qPCR was performed using SYBR Green Low ROX qPCR Mix (Thermo Scientific #AB-1323A) and analyzed using a QuantStudio 6 Flex Real-Time PCR system. Gene normalization was performed using 18S, and ROX was the internal control. The Relative Quantification (RQ) of gene expression was calculated using the following formula: 2^(−ddCT)^, where ddCT is the difference between the dCT(gene of interest) and the dCT(control gene). The dCT(gene of interest) is the average Ct value (of three technical replicates) of the gene of interest minus the average Ct value of 18S in that sample. 18S was used as the control gene for all experiments except for those in which cells were treated with saquinavir. In these experiments, GAPDH was used as the control. The following primers were used as described in (80): F: 5′ CTCTGCTCCTCCTGTTCGAC 3′; R: 5′ AGTTAAAAGCAGCCCTGGTGA 3′. An ordinary one-way ANOVA was used to compare conditions. Turkey’s multiple comparisons test, with a single pooled variance, was used to derive *P* values.

### Additional drug treatments

Saquinavir treatment of ACH2 cells was conducted as described above (Fig. 2A) plus the addition of 5 µM saquinavir (MedChemExpress, HY-17007) at time zero, along with the initial MLN treatment. Saquinavir was not replenished after the initial dose. When measuring RNA expression in cells treated with Saquinavir, GAPDH was used as the control in place of 18S for RT-qPCR. JSH-23 (TargetMol, #749886-87-1) and BMS-345541 (TargetMol, #445430-58-0) were added at the concentration noted in figure legends at time zero, along with initial MLN treatment, and not replenished.

### Evaluation of HIV msRNA in CD4+ T cells from ART-suppressed PLWH (TILDA)

Frozen, banked PBMCs were selected from the RADAR cohort at Northwestern University (52). Donors who had been on ART for >3 years or who seroconverted during the study (and thus were immediately put on medication) were undetectable at the time of sample collection (<40 copies/mL), and self-reported good adherence to the ART regimen and lacked substance use were selected.

PBMCs were thawed and rested at 37°C for 2 hours prior to negative selection of CD4+ T cells using the EasySep Human CD4+ T-cell Isolation Kit (StemCell Technologies, #17952). Cells were seeded at a density of 2 × 10^6^ cells/mL and rested in complete media for an additional 3 hours prior to cellular activation with 500× PMA and ionomycin (eBioscience Cell Stimulation Cocktail, #00-4970-93) or DMSO control for a total of 12 hours. After 4 hours, non-stimulated and stimulated cells were divided in half and treated with either DMSO (control) or 200 nM of MLN.

After 12 hours of stimulation, cells were washed and pelleted in a benchtop centrifuge, and *tat/rev* msRNA was measured according to the previously defined TILDA v2.0 protocol (51). In brief, cells were diluted to 3 × 10^5^–1×10^5^ cells/mL in RPMI. From each aliquot, 10 µL of the cell suspension was distributed to 24 wells of a 96-well plate containing 2 µL One-Step RT-PCR enzyme (Qiagen, #210212), 10 µL 5× One-Step buffer, 10 µL 0.3% Triton-x (Fisher BP151-500), 0.25 µL 40 U/µL RNAsin (Promega N2111), 2 µL dNTPs (10 mm each), 1 µL 20 µM forward primers (tat1.4 5′ TGGCAGGAAGAAGCGGAG 3′, 1 µL 20 µM reverse primer (rev 5′ GGATCTGTCTCTGTCTCTCTCTCCACC 3′), and nuclease-free water (50). To increase sensitivity, for donors B and C, RNA was extracted first using the Qiagen RNeasy Mini Kit (#74106) from each of the 24 wells individually, and 10 µL of RNA was added to the one-step PCR. One-step RT-PCR was run on a thermocycler using the following conditions: 50°C for 30 min, 95°C for 15 min, 25 cycles of 95°C for 30 s, 55°C for 1 min, 72°C for 2 min followed by 72°C for 5 min. After reverse transcription, 2 µL from each of the 24 wells was pooled across each condition and run on the QuantStudio Absolute Q Digital PCR System to give an absolute quantification of msRNA. 1 µL of sample was added to 2 µL Absolute Q 5X Master Mix (Applied Biosciences), 5 µM Probe HIV, 20 µM tat2.0, 20 µM reverse primer, and dH_2_O, and run with the following cycling conditions: 45 cycles of 95°C for 23 s, followed by 30 s at 60°C (50).

### p24 ELISA

Supernatant from ACH2 cells was collected and clarified by either spinning down and discarding the pellet or by straining through a 0.45 µm filter. Extracellular p24 capsid protein was measured in triplicate via sandwich ELISA using commercially available Alliance HIV-1 P24 Antigen Kit (NEK050B001KT) according to the manufacturer’s protocol. An ordinary one-way ANOVA was used to compare conditions. Turkey’s multiple comparisons test, with a single pooled variance, was used to derive *P* values.

### Infectivity assays

ACH2 cells were treated with LRAs and MLN as described in Fig. 2A. ACH2 cell supernatant was collected 48 hours after LRA reactivation and normalized for virus content (as determined by p24 ELISA). To test the infectivity of these viruses, the TZM-bl reporter cell line was plated at a density of 10,000 cells/well in a 96-well plate. 24 hours later, the media was replaced, and cells were infected with equivalent amounts of virus from ACH2 cell supernatant in triplicate wells. The following morning, the media was replaced, and cells were incubated for another 24 hours. 48 hours post-infection, cell luminescence was measured using the Britelite plus Reporter Gene Assay System (Perkin Elmer, #50-209-9084) according to the manufacturer’s protocol. Infectivity was determined based on the relative luciferase activity downstream of the HIV LTR in TZM-bl cells. Infectivity was calculated by normalizing the average relative luminescence units (RLU) to the DMSO control. Relative infectivity was calculated by setting the LRA-only control to 100% infectivity and calculating the percent change in infectivity compared to this condition. An ordinary one-way ANOVA was used to compare conditions. Turkey’s multiple comparisons test, with a single pooled variance, was used to derive *P* values.

## Data Availability

The raw data for each figure are available in accordance with Northwestern University policies at an open Northwestern data repository: https://prism.northwestern.edu/communities/daquilalab. Data can be found at the following DOI: 10.18131/ptcgp-kq338.
